# Comprehensive analysis of T-cell receptor repertoire in patients with acute coronary syndrome by high-throughput sequencing

**DOI:** 10.1186/s12872-020-01538-6

**Published:** 2020-05-27

**Authors:** Sudong Liu, Zhixiong Zhong, Wei Zhong, Ruiqiang Weng, Jing Liu, Xiaodong Gu, Yongyu Chen

**Affiliations:** 1 Clinical Core Laboratory, Meizhou People’s Hospital (Huangtang Hospital), Meizhou Hospital Affiliated to Sun Yat-sen University, No 63 Huangtang Road, Meijiang District, Meizhou, 514031 P. R. China; 2 Guangdong Provincial Key Laboratory of Precision Medicine and Clinical Translational Research of Hakka Population, Meizhou, 514031 P. R. China; 3 Center for Precision Medicine, Meizhou People’s Hospital (Huangtang Hospital), Meizhou Hospital Affiliated to Sun Yat-sen University, Meizho, 514031 P. R. China; 4 Center for Cardiovascular Diseases, Meizhou People’s Hospital (Huangtang Hospital), Meizhou Hospital Affiliated to Sun Yat-sen University, Meizhou, 514031 P. R. China

**Keywords:** Acute coronary syndrome (ACS), T-cell receptor (TCR), Clonotype, High-throughput sequencing (HTS)

## Abstract

**Background:**

This study aims to investigate the T-cell receptor (TCR) repertoire in patients with acute coronary syndrome (ACS).

**Methods:**

The TCR repertoires of 9 unstable angina patients (UA), 14 acute myocardial infarction patients (AMI) and 9 normal coronary artery (NCA) patients were profiled using high-throughput sequencing (HTS). The clonal diversity of the TCR repertoires in different groups was analyzed, as well as the frequencies of variable (V), diversity (D) and joining(J) gene segments.

**Results:**

ACS patients including UA and AMI, showed reduced TCRβ diversity than NCA patients. ACS patients presented higher levels of clonal expansion. The clonotype overlap of complementarity determining region 3(CDR3) was significantly varied between different groups. A total of 10 V genes and 1 J gene were differently utilized between ACS and NCA patients. We identified some shared CDR3 amino acid sequences that were presented in ACS but not in NCA patients.

**Conclusions:**

This study revealed the distinct TCR repertoires in patients with ACS and demonstrated the presence of disease associated T-cell clonotypes. These findings suggested a role of T cells in ACS and provided a new way to explore the mechanisms of ACS.

## Background

Acute coronary syndrome (ACS) is the severe form of coronary artery disease (CAD), which remains the leading cause of morbidity and mortality worldwide [1]. It has been well documented that atherosclerosis, the major cause of CAD, is a chronic inflammatory process involving a variety of innate and adaptive immune components [2, 3]. T cells are prominent component in the atherosclerotic plaque, and typical signs of T cell activation were observed in the blood of patients with ACS [4–6]. Evidence suggested that ACS patients had higher frequency and enhanced function of T cells compared to patients with stable angina (SA) [7]. Therefore, T cells mediated adaptive immunity may play an important role in the pathological process of ACS.

Atherosclerosis which is featured by the formation of plaques containing immune cells, foam cells and others components is suggested as the most important pathogenic mechanism of ACS [8]. Previous study has found that T cells accumulated in human atherosclerotic plaques, most of which were effector and memory T cells [9]. Meanwhile, researchers have demonstrated the existence of T cells within the aortic adventitia of vessels [10]. Accumulating evidences suggest that T cells play an important role in the immunological mechanisms of the ACS [11, 12]. However, the clonotype feature of involved T cells remained unclarified.

T cell receptor (TCR) expressed on the surface of T cells recognizes the antigenic peptides presented by antigen presented cells (APC). Human TCRs are commonly composed of α and β chains [13]. TCRα chains consist of variable (V), joining (J) and constant (C) regions, while TCRβ chains consist of V, J, C and diversity (D) regions. The diversity of TCR is generated by rearrangement of the V, D, J and C regions. Random insertions and deletions of nucleotides at the junctions increase the diversity [14]. The alteration of TCR repertoire has been observed in many diseases, such as cancer, autoimmune disorders and infectious diseases [15, 16]. Thus, TCR repertoire has great diagnosis value and clinical utility, as its diversity reflects the state of immune system [17]. Researchers begin to explore the association between changes in TCR repertoire and diseases, on propose to identify novel biomarkers or prognostic factors [18, 19].

In the present study, we aimed to characterize the TCR repertoire in peripheral blood samples from ACS patients using high-throughput sequencing. We analyzed the diversity of the TCR repertoire, V/J gene utilization and disease associated TCRβ clonotypes. The present study might contribute to the understanding of the roles of T cells in ACS development and provides a new way to explore the mechanisms of ACS.

## Methods

### Subjects

Patients were enrolled from Cardiology Department of Meizhou People’s Hospital (Huangtang Hospital), Meizhou Academy of Medical Sciences, Meizhou Hospital Affiliated to Sun Yat-sen University, Meizhou, China. Patients were diagnosed as UA if they had: (1) angina-like chest pain or ischemic equivalent; (2) electrocardiographic abnormalities compatible on at least two contiguous leads; (3) at least one major pericardial vessel with > 70% stenosis. Patients were diagnosed as AMI if met the above 3 criterions plus another one: (4) abnormalities above the upper normal limit for myocardial necrosis biomarkers (troponin and/or CKMB). Normal coronary artery (NCA) patients were defined as no stenosis in coronary arteries by quantitative coronary angiography and served as controls in this study. Patients were excluded if they had the following manifestations or diseases, i.e. left ventricular ejection fraction ≤45%, congestive heart failure, chronic kidney or hepatic disease and cancer. The diagnosis was made by two senior cardiologists. This study was approved by the Ethics Committee of the Meizhou People’s Hospital (Huangtang Hospital). Each patient had signed a written informed consent.

### Sample collection

Peripheral venous blood samples were collected and placed in EDTA-coated tubes. The blood samples were processed within 30 min. PBMCs were isolated from fresh whole blood by density gradient centrifugation using Hypaque-Ficoll (GE Healthcare Bio-sciences AB, Sweden). The isolated PBMCs were lysed using TRIzol reagent (Invitrogen, USA) and stored at − 80 °C until used.

### RNA extraction and cDNA synthesis

Total RNA was extracted from 1 × 10^6^ PBMCs from each sample using RNeasy Mini Kit (QIAGEN, German) following the manufacture’s protocol. The concentrations and purity of RNA were measured by Nanodrop − 1000 spectrophotometer (Thermo FisherScientific Inc., USA).RNA integrity was analyzed by the Bioanalyzer 2100 system (Agilent Technologies, USA). cDNA libraries were prepared by 5′-rapid amplification of cDNA ends (RACE) using the SMARTer PCR cDNA synthesis kit (Clontech, USA) as described previously [20]. Briefly, 1.5 μg of total RNA was mixed with the primer BC1R (CAGTATCTGGAGTCATTGA)(20 μM) in a sterile thin-walled reaction tube. The tube was placed on a thermal cycler and incubated for 3 min at 70 °C and then for 2 min at 42 °C to anneal synthesis primer. A reaction mix containing first strand buffer, 5′-template switch adapter and SMARTScribe reverse transcriptase was prepared and added to the tube. The tube was incubated at 42 °C for 60 min.

### Library construction and sequencing

Libraries were prepared using two-round PCR with specific primers as reported previously [20]. Briefly, in the first-run PCR amplification, the reaction system containing cDNA, Advantage 2 polymerase mix (Clontech, USA), universal primer smart 20 (CACTCTATCCGACAAGCAGTGGTATCAACGCAG), and TCRβ specific primer (TGCTTCTGATGGCTCAAACAC) was prepared. TCRβ was amplified using LightCycler 480 (Roche, USA) with the following program: 95 °C for 20 s, 65 °C for 20 s, 72 °C for 50 s, for a total of 18 cyclers. The products were purified using the QIAquick PCR purification kit (Qiagen, German).

In the second-run PCR, the reaction system was prepared containing the purified products, universal primers Step1(CACTCTATCCGACAAGCAGT), specific primer hum-bcj (ACACSTTKTTCAGGTCCTC). Amplification was carried out on LightCycler 480 with the following program: 95 °C for 20 s, 65 °C for 20 s, 72 °C for 50 s, for a total of 12 cycles. The products were purified as above. Libraries were amplified using Illumina sequencing primers with barcodes. Then, paired-end 150 bp sequencing was performed on the Illumina HiSeq2000 platform in ShenZhen Realomics Inc.

### Bioinformatic analysis

The sequencing quality of the library by Illumina Hiseq 2000 was evaluated by the Realomics system formula. Briefly, the adaptor reads and the low-quality reads were filtered to obtain clean data. Subsequently, the clean data was aligned to human IGH database and analyzed by miXCR [21]. The high-quality reads were further assembled into clonotypes, correcting for PCR and sequencing errors using a heuristic multilayer clustering by VDJ tool [22, 23].

### Statistical analysis

The data were analyzed with IBM SPSS 20.0 software (Social Science Statistics Software Package). Continuous data were presented as means ± standard deviation (SD) and categorical variables were presented as number (%). Data were assessed for their normality using Kolmogorov–Smirnov test. One-way analysis of variance (ANOVA) was used for normally distributed data, and Wilcoxon rank-sum test was used for data did not follow a normal distribution. For the categorical variables, Chi-square test was performed. *P* <  0.05 was considered statistically significant.

## Results

### Profile of TCRβ CDR3 in ACS patients

Our study cohort consisted of 9 NCA, 9 UA and 14 AMI patients. The clinical characteristics of participants are shown in Table 1. There is not significant difference in age and gender, as well as lipid levels between different groups. As for medicine use, UA and AMI patients take more statins, ACEI/ARB and β-blocker than NCA patients.

**Table 1:** Clinical Characteristics of study subjects for this study

Variables	NCA (*n* = 9)	UA (*n* = 9)	AMI (*n* = 14)	***P*** value^a^
Age, years	55.4 ± 6.95	56.1 ± 5.46	59.86 ± 6.76	0.28
Gender (F/M)	5/4	5/4	8/6	0.99
Current smoking, n (%)	2(22.2%)	2(22.2%)	4(28.6%)	0.91
Triglycerides, mmol/L	1.36 ± 0.56	1.71 ± 0.56	1.98 ± 1.19	0.49
Cholesterol, mmol/L	4.85 ± 1.00	5.26 ± 1.23	4.69 ± 1.48	0.58
HDL, mmol/L	1.25 ± 0.27	1.25 ± 0.24	1.11 ± 0.24	0.30
LDL, mmol/L	2.66 ± 0.62	3.01 ± 0.76	2.64 ± 0.80	0.47
Medicine use				
Nitrate esters drug, n (%)	0	1(11.1%)	0	0.26
Clopidogrel, n (%)	1(11.1%)	5(55.6%)	5(35.7%)	0.08
Statins, n (%)	0	6(66.7%)	1(7.1%)	< 0.01
Aspirin, n (%)	2(22.2%)	7(77.8%)	6(42.9%)	0.06
ACEI/ARB, n (%)	0	4(44.4%)	1(7.1%)	0.01
Ca_2_^+^ antagonist, n (%)	0	2(22.2%)	0	0.06
β - blocker, n (%)	1(11.1%)	6(66.7%)	0	< 0.01

*HDL* High-density lipoprotein;

*LDL* Low-density lipoprotein;

*ACEI/ARB* Angiotensin-converting enzyme inhibitors/angiotensin antibody;

^a^comparison between three groups using one-way ANOVA or Chi-square

We obtained a total number of 459,866,341 clean reads, with an average of 14,370,823 for each sample. We identified 51 distinct Vβ gene segments and 14 distinct Jβ gene segments among the participants. No difference was observed in the TCRβ V gene composition between different groups (Supporting Fig. S1). A detailed description of sequencing data including total clean reads, clones, unique V genes and other information are presented in Supporting Table S1.

### A reduced TCRβ CDR3 diversity in ACS patients

**Fig. 1:**
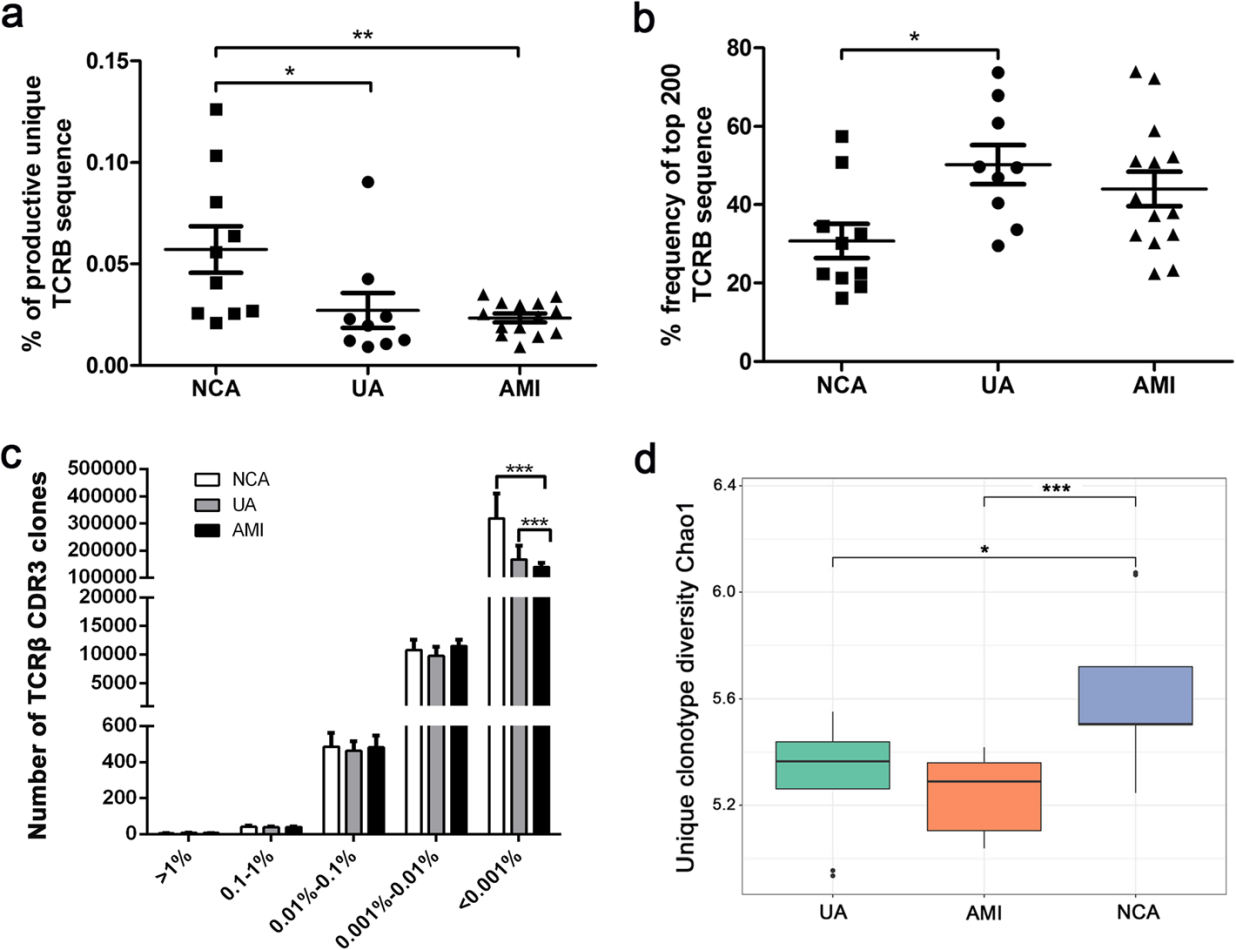
Clonal distribution of T cells in patients with UA, AMI and NCA. **a** Frequency of unique TCR β sequences identified in the peripheral blood of patients with UA, AMI and NCA. Differences between groups were compared using one-way ANOVA. **b** Percentage frequency of top 200 TCRβ nucleotide sequences in UA, AMI and NCA. Differences between groups were compared using one-way ANOVA. **c** Frequency distributions of TCRβ CDR3 clones from UA(*n* = 9), AMI(*n* = 14) and NCA(n = 9) patients. Differences between groups were compared using one-way ANOVA. **d** Diversity metrics for TCRβ CDR3 repertoires in UA, AMI and NCA. Data were compared using Wilcoxon rank-sum test. *P < 0.05, **P < 0.01, ***P < 0.001

The percentage of the productive unique TCRβ sequence provides a general assessment of sample diversity. As shown in Fig.1a, the percentage of the productive unique TCRβ sequence in AMI patients(2.35% ± 0.82%) and UA patients(3.08% ± 2.59%) were both significantly lower than that in NCA (5.70% ± 3.65%), but there was no significant difference between AMI and UA. The expansion level of each unique clone was another major measurement for immune diversity. Clonal expansion was assessed by cumulative percentage of the repertoire. The average fraction of the top 200 TCRβ sequences was 30.72% in NCA, 51.47% in UA, and 44.02% in AMI, suggesting a clonal expansion of TCRβ nucleotide sequences in UA and AMI patients (Fig. 1b). The number of TCRβ CDR3 clones were similar between different groups, except for the low frequent clones (Fig. 1c). Finally, the diversity of TCRβ CDR3 was assessed by Chao 1 approach. It was suggested that diversity of T cell clones significantly decreased in AMI and UA patients as compared to NCA (Fig. 1d).

### The usage patterns of Vβ and Jβ gene segments in ACS patients

**Fig. 2:**
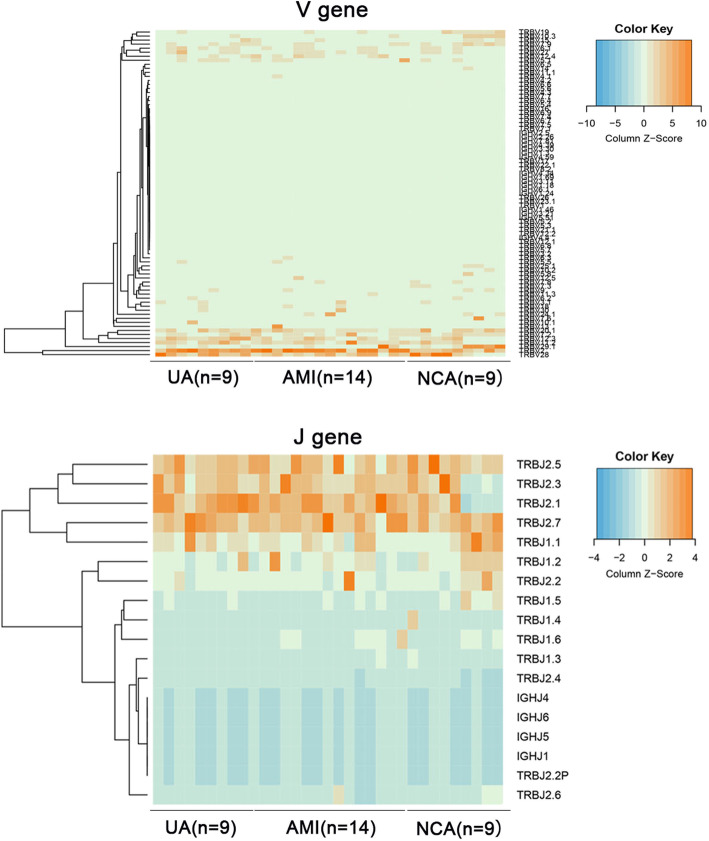
Heatmap of Vβ and Jβ gene segments usage in samples from UA, AMI and NCA. The heatmap bar indicates the usage frequency of Vβ or Jβ gene segments in each sample

**Fig. 3:**
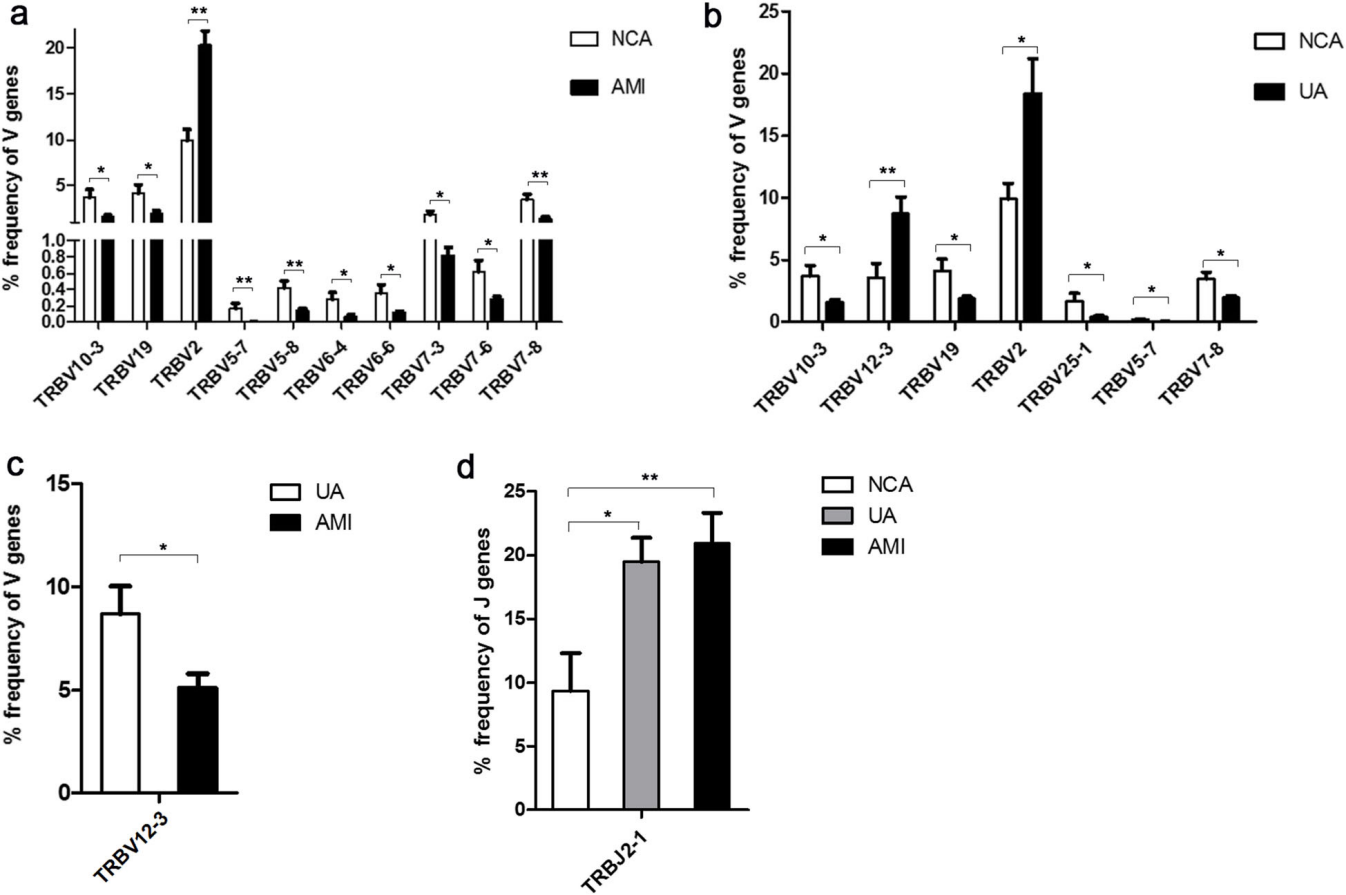
Vβ and J β gene usage of clonotypes in patients with UA, AMI and NCA. The frequencies of Vβ and Jβ genes were calculated and compared between AMI, UA and NCA. **a** Vβ genes with different frequencies between AMI and NCA; **b** Vβ genes with different frequencies between UA and NCA; **c** Vβ genes with different frequencies between AMI and UA; **d** Jβ gene with different frequencies between AMI, UA and NCA. Data were compared using Wilcoxon rank-sum test. *P < 0.05, **P < 0.01, ***P < 0.001

The frequency heatmap of Vβ and Jβ genes showed the distribution patterns of Vβ and Jβ in NCA, UA and AMI patients (Fig. 2 and Supporting Fig. S2). Frequencies of most Vβ and Jβ genes were similar between different groups, apart from some Vβ and Jβ gene. In AMI, 10 Vβ clonotypes were differentially used compared with NCA, and all of them showed lower frequencies except TRBV2 (Fig. 3a). In UA, TRBV12–3 and TRBV2 were more frequent than in NCA, while TRBV10–3, TRBV19, TRBV25–1, TRBV5–7 and TRBV7–8 were less frequent (Fig. 3b). In AMI patients, TRBV12–3 was more frequent than in UA (Fig. 3c). In addition, TRBJ2–1 was more frequent in UA and AMI than that in NCA (Fig. 3d).

### TCRβ clonotype overlap within and between different groups

**Fig. 4:**
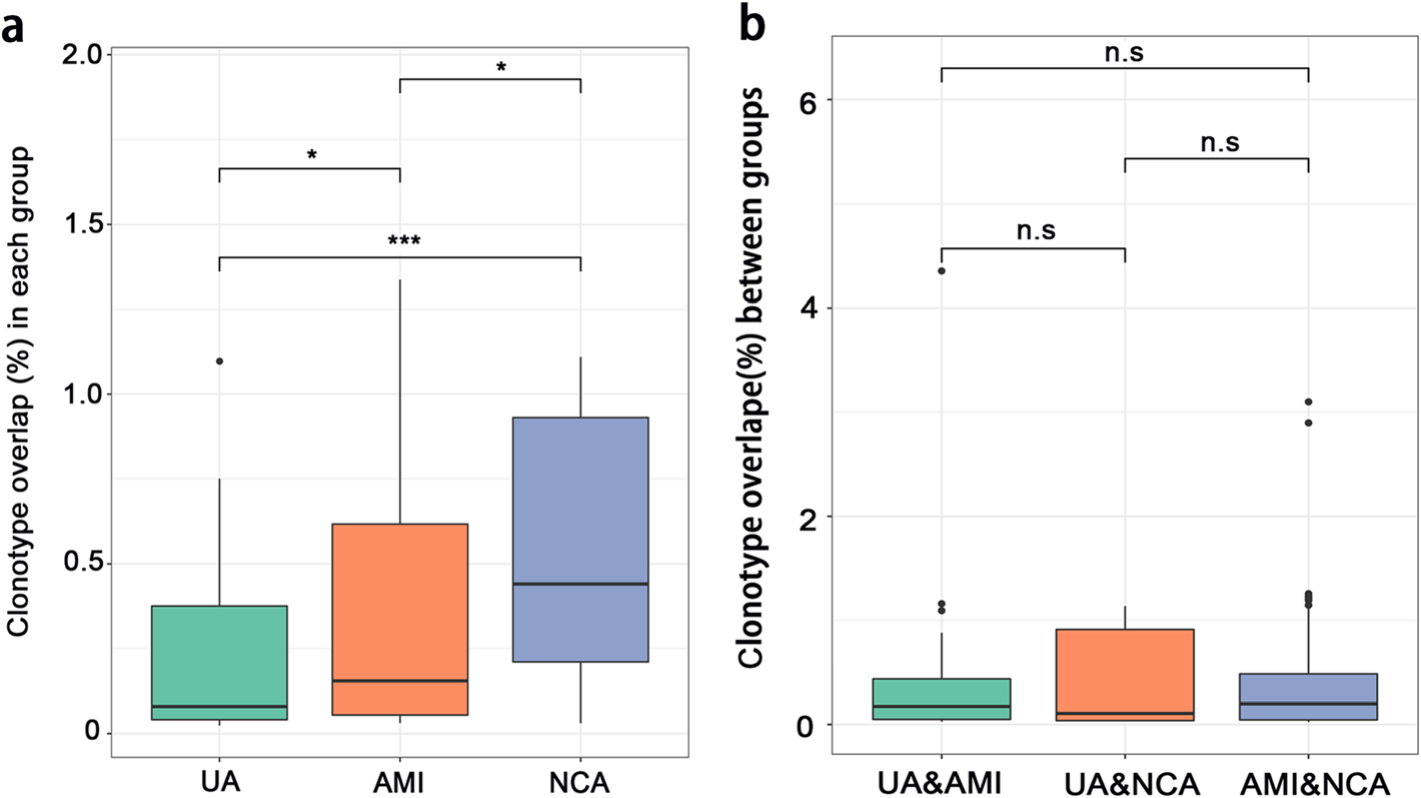
Overlap in T-cell specificity in each group and between disease groups. **a** Data show the overlap of clonotypes in each disease group, UA, AMI and NCA. **b** Data show the overlap of clonotypes between disease groups, UA and AMI, UA and NCA, AMI and NCA. Data were compared using Wilcoxon rank-sum test. *P < 0.05, **P < 0.01, ***P < 0.001

We next investigated TCRβ clonotype overlap in patients of the same group and TCRβ clonotype overlap between different groups. The average TCRβ clonotype overlap in UA and AMI patients were significantly lower than that in NCA patients (*P* <  0.001 and *P* <  0.05). The average TCRβ clonotype overlap in UA patients was lower than that in AMI patients (*P* <  0.05) (Fig. 4a). However, the TCRβ clonotype overlap between two groups remained very low, and did not show any difference (Fig. 4b). Collectively, these data suggested a greater sharing of TCRβ repertoire in NCA patients in compared to the AMI and UA patients.

### Presence of disease-associated clonotypes in UA and AMI patients

To determine the immune response characteristics specific to ACS, we searched for TCRβ clonotypes that were presented in UA or AMI patients but NCA patients. We identified 3 TCRβ CDR3 clonotypes that were frequent in UA patients but absent in NCA patients. There were 9 TCRβ CDR3 clonotypes that were frequent in AMI patients but rare in NCA patients. In addition, we found 5 TCRβ CDR3 clonotypes that were frequently shared in both UA and AMI patients, while scarcely presented in NCA patients (Table 2). A total of 28 TCRβ CDR3 clonotypes were specifically frequent in AMI patients, and additional 30 TCRβ CDR3 clonotypes were specifically frequent in UA patients (Supporting Table S2 and S3). These data suggested the presence of disease-associated TCRβ CDR3 clonotypes in UA and AMI.

**Table 2:** Shared TCRβ CDR3 clonotypes in ACS patients

TCRβ CDR3	NCA		UA	AMI		
	Case	Frequency	Case	Frequency	Case	Frequency
**UA**						
CASSRTSGGYNEQFF	0/9	NA	8/9	5.94E-07	8/14	1.07E-06
CASSAGRETQYF	0/9	NA	7/9	4.64E-07	6/14	1.26E-05
CASSLTSGLYNEQFF	0/9	NA	7/9	5.59E-06	3/14	9.51E-07
CASSPSGGQETQYF^a^	1/9	3.52E-08	7/9	7.50E-06	10/14	3.63E-06
**AMI**						
CASSLGGRALEQFF	1/9	3.25E-08	4/9	6.83E-07	11/14	6.14E-05
CASSKTSGRAFEQFF	1/9	3.25E-08	3/9	4.53E-07	10/14	4.01E-07
CATSRDGGVTNQYF	1/9	1.62E-08	4/9	3.19E-05	10/14	2.30E-07
CASSVPLRLAESSYNEQFF	1/9	7.77E-08	4/9	1.49E-06	10/14	2.63E-04
CASSTGQEQYF	1/9	2.20E-07	3/9	2.72E-07	10/14	3.41E-05
CASSPHDQETQYF	1/9	1.10E-07	4/9	5.78E-07	10/14	2.64E-07
CASSPQGEVGYTF	1/9	1.62E-08	4/9	2.46E-07	10/14	1.93E-07
CASRPGRGPDTQYF	1/9	1.55E-08	4/9	3.61E-07	10/14	2.48E-07
CASSPSGGQETQYF^a^	1/9	3.52E-08	7/9	7.50E-06	10/14	3.63E-06
**UA&AMI**						
CASSPSGGQETQYF^a^	1/9	3.52E-08	7/9	7.50E-06	10/14	3.63E-06
CASSLSGGSYNEQFF	3/9	2.78E-06	7/9	2.60E-08	10/14	2.19E-07
CASSYSYNEQFF	3/9	3.80E-08	7/9	4.08E-06	10/14	4.54E-06
CASRDRGSTDTQYF	3/9	5.73E-07	7/9	5.49E-04	10/14	4.80E-07
CASSQTNQETQYF	3/9	1.26E-07	7/9	3.23E-06	10/14	6.76E-07

^a^The same sequence

## Discussion

Although modern medical treatment has considerably improved the outcome of ACS, it is still the deadliest disease all around the world [24]. In this study, we comprehensively analyzed the TCR CDR3β repertoire of patients with UA, AMI and NCA. The results suggested that: (1) diversity of T cell clones was reduced in patients with UA and AMI; (2) the Vβ and Jβ genes usage patterns differed in UA, AMI and NCA; (3) disease-associated TCR CDR3β clonotypes were identified.

T cell-mediated immune responses are principal component of cellular immune response and protected against many diseases and infections [25, 26]. Previous studies have found a higher level of activated T-cells in ACS patients as compared with stable angina patients [5, 6]. ApoE^−/−^ mice lacking CD4+ T cells were found less susceptible to atherosclerosis compared with wild-type mice, and transfer of CD4+ T cells to immunodeficient ApoE^−/−^ mice significantly promoted atherosclerosis. These findings suggested a proatherosclerotic role of CD4+ T cells. The function of CD4+ T cells was proved exerted by production of IFN-γ [27, 28]. Another study focused on the TCR γδ T repertoire suggested that clonal expansion of γδ T cell and altered expression of IL-17A was associated with the clinical outcome of AMI [29]. Regulatory T cell (Treg) has been shown to induce regression of atherosclerosis and to increase plaque stability in mice [30]. Treg produced anti-inflammatory cytokines such as IL-10 and TGFβ, thus prevented disease progression of atherogenesis [31]. Another study showed that the dysregulation of helper T cells impacted the immune response and impaired the stability of plaque in ACS patients [7]. However, there are still many unsolved problems regarding the T cells in procession of ACS.

The CDR3 region represents the uniqueness of each TCR and thus used as TCR signature or barcode [32]. Diversified TCRs were required for an intense adaptive immune responses, while pressures from both internal and external help shape the diversity of TCR [33, 34]. The TCR repertoire of patients with atherosclerosis has been investigated and level of diversity was reduced in AS plaques [35]. In the present study, we found that clonal diversity of the TCR CDR3 β decreased in ACS patients as compared to NCA. Meanwhile, a higher percentage of frequent clones (the top 200 TCRβ) were observed in ACS patients, especially in UA. Additionally, we found that the average overlap between ACS and NCA was significantly different. Collectively, these data reflected that clonal expansion of T cells occurred in ACS patients.

Many local autoantigens contribute to these immunoregulatory abnormalities, resulting in the clonal restriction of T cells [36]. This restriction is associated with the plaque instability and inflammatory response [37]. Researchers have found oxidative low density lipid (ox-LDL) specific T cells and oligoclonal T cells in atherosclerotic lesions [38]. T cells expressing Vβ 6 were significantly proliferated in atherosclerotic plaques and responsible for ox-LDL recognition [39]. In our study, we observed that frequencies of 11 V gene segments and 1 J gene segment were altered in ACS patients compared to NCA.

The numbers and types of T cell clones containing different TCR amino acid/nucleic acid sequences are extremely large. Many techniques used in most of the previous studies are difficult to accurately reveal the features of TCRβ CDR3 clonotypes [40]. The immune repertoire sequencing technology used in our study is based on the multiplex PCR and next-generation sequencing, and much sensitive than previous method [41]. Meanwhile, we characterized the receptor signatures of T cells from ACS patients without any in vitro manipulation, thus avoided bias and obtained a near in vivo result.

## Conclusions

In summary, the present study investigated a comprehensive characterization of TCRβ CDR3 repertoire in patients with UA, AMI and NCA. Our data suggested T cell diversity was significantly altered in ACS compared with NCA, and we identified some shared TCRβ clonotypes which were disease-associated. These results facilitate to improve our understanding of ACS and provide a new sight to explore the mechanism of this disease.

## Supplementary information

**Additional file 1: Figure S1.** Numbers of TRBV gene segments used in patients with UA, AMI and NCA

**Additional file 2: Figure S2.** Vβ gene usage of clonotypes in the patients with UA, AMI and NCA. Data show the percentage frequency of V genes used by clonotypes in the patients with UA, AMI and NCA.

**Additional file 3: Figure S3.** Jβ gene usage of clonotypes in the patients with UA, AMI and NCA. Data show the percentage frequency of J genes used by clonotypes in the patients with UA, AMI and NCA.

**Additional file 4: Table S1.** Detailed immune repertoire sequencing data. **Table S2.** Frequent unique TCRβ CDR3 aa clonotypes in AMI patients. **Table S3.** Frequent unique TCRβ CDR3 aa clonotypes in UA patients.

## Data Availability

The datasets used and/or analyzed during the current study available from the corresponding author on reasonable request.

## Abbreviations

ACS: Acute coronary syndrome
ANOVA: One-way analysis of variance
CAD: Coronary artery disease
CDR3: Complementarity determining region 3 (CDR3)
HTS: High-throughput sequencing
NCA: Normal coronary artery
PBMC: Peripheral blood mononuclear cell
SA: Stable angina
TCR: T-cell receptor
