# Supplementary figures and images for: Comprehensive analysis of T-cell receptor repertoire in patients with acute coronary syndrome by high-throughput sequencing
Source: BMC Cardiovasc Disord. 2020 May 27;20:253. doi: 10.1186/s12872-020-01538-6 (PMC7254720; doi:10.1186/s12872-020-01538-6)

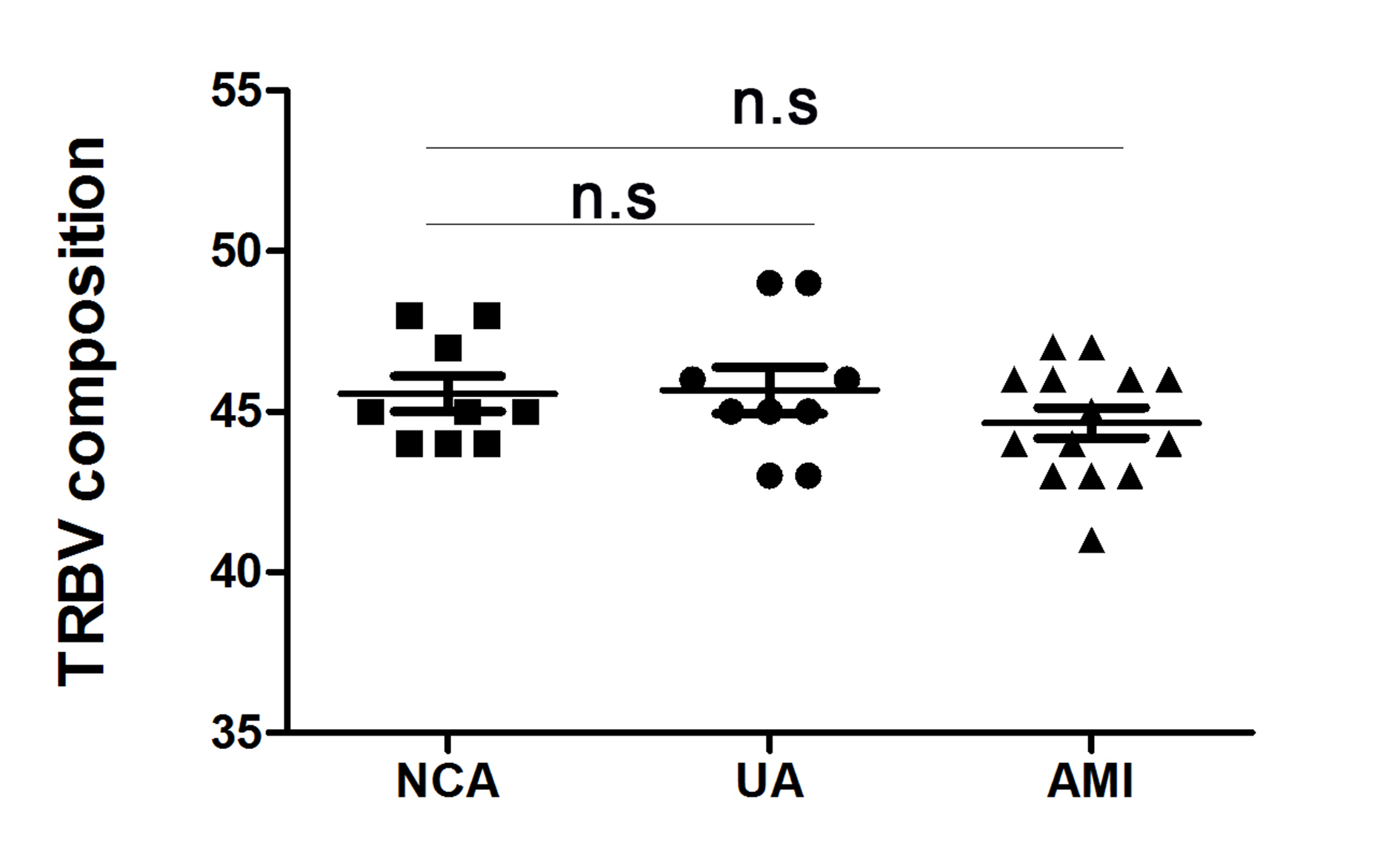

Supplement: Supplementary file 1 — Additional file 1: Figure S1. Numbers of TRBV gene segments used in patients with UA, AMI and NCA [file 12872_2020_1538_MOESM1_ESM.tif]

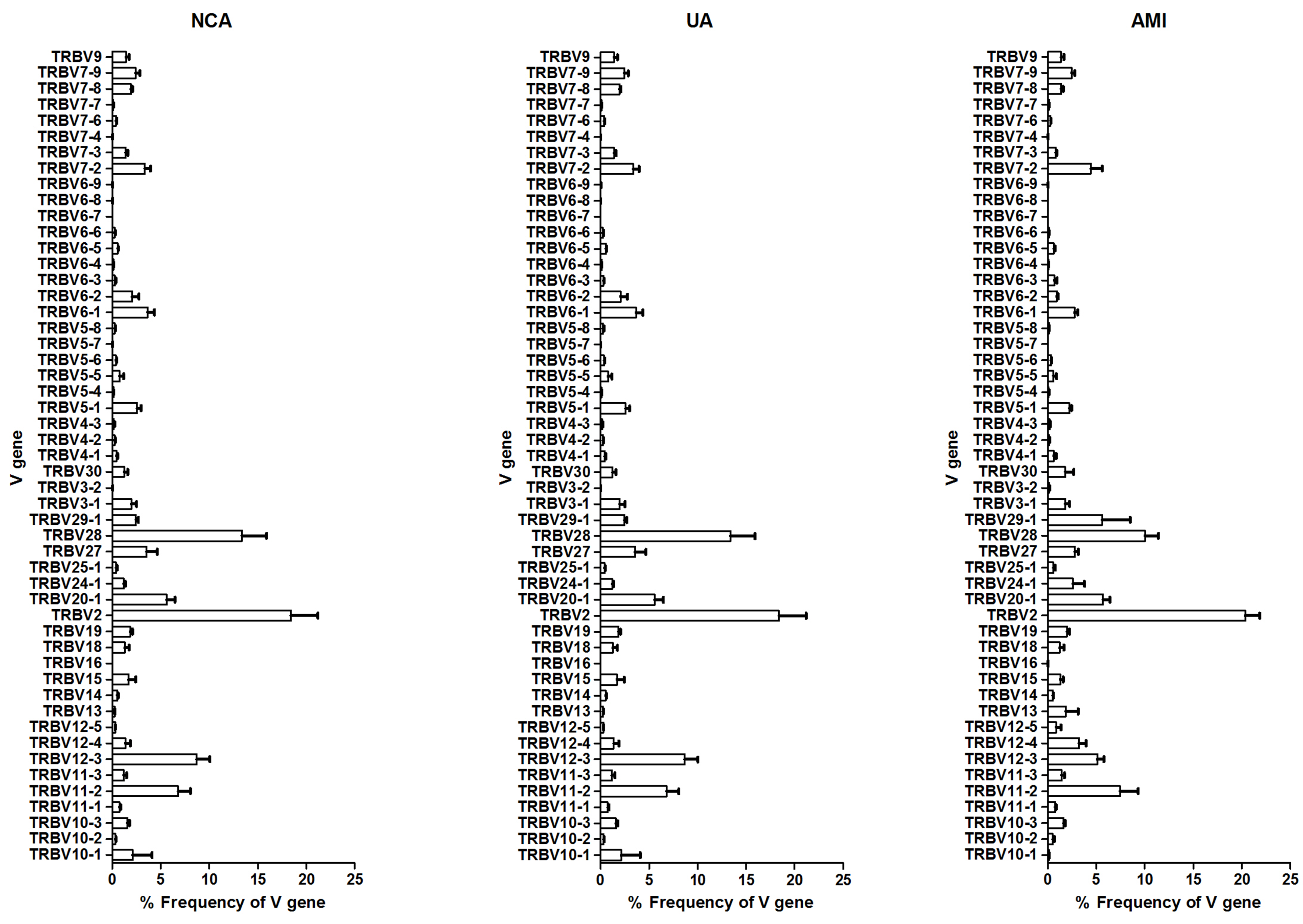

Supplement: Supplementary file 2 — Additional file 2: Figure S2. Vβ gene usage of clonotypes in the patients with UA, AMI and NCA. Data show the percentage frequency of V genes used by clonotypes in the patients with UA, AMI and NCA. [file 12872_2020_1538_MOESM2_ESM.tif]

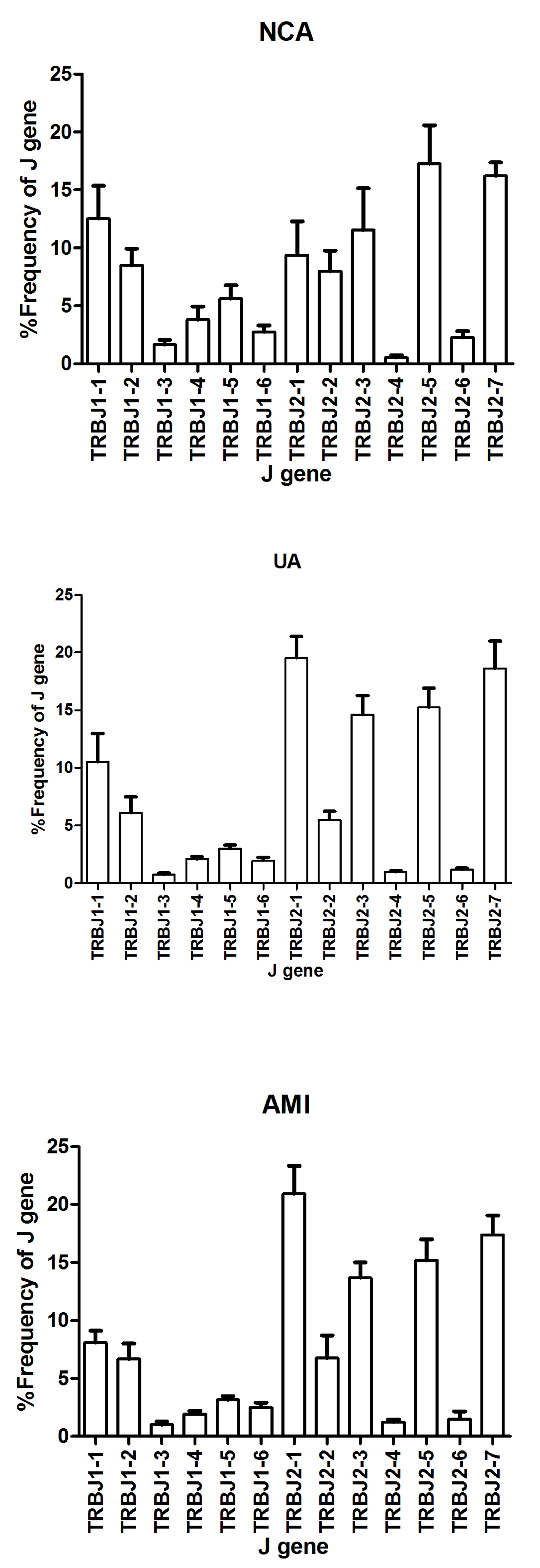

Supplement: Supplementary file 3 — Additional file 3: Figure S3. Jβ gene usage of clonotypes in the patients with UA, AMI and NCA. Data show the percentage frequency of J genes used by clonotypes in the patients with UA, AMI and NCA. [file 12872_2020_1538_MOESM3_ESM.tif]
