# Supplementary material for: Comprehensive analysis of T-cell receptor repertoire in patients with acute coronary syndrome by high-throughput sequencing
Source: BMC Cardiovasc Disord. 2020 May 27;20:253. doi: 10.1186/s12872-020-01538-6 (PMC7254720; doi:10.1186/s12872-020-01538-6)
Supplement: Supplementary file 4 — Additional file 4: Table S1. Detailed immune repertoire sequencing data. Table S2. Frequent unique TCRβ CDR3 aa clonotypes in AMI patients. Table S3. Frequent unique TCRβ CDR3 aa clonotypes in UA patients. [file 12872_2020_1538_MOESM4_ESM.docx]

**Supporting Table S1 Detailed immune repertoire sequencing data.**

| **Patient** | **Status** | **Read**  **(Pair)** | **Clone** | **Clone**  **(%)** | **Unique V** | **Unique J** | **Unique VJ** | **Unique**  **CDR3aa** | **Unique**  **CDR3nt** |
| --- | --- | --- | --- | --- | --- | --- | --- | --- | --- |
| P1 | NCA | 15232794 | 7347548 | 48.24 | 44 | 13 | 444 | 226878 | 242062 |
| P2 | NCA | 12825739 | 6237619 | 48.63 | 44 | 13 | 483 | 455340 | 502765 |
| P3 | NCA | 12091563 | 5847104 | 48.35 | 47 | 13 | 494 | 224670 | 238550 |
| P4 | NCA | 12731367 | 6164610 | 48.42 | 45 | 13 | 459 | 156794 | 166407 |
| P5 | NCA | 13619181 | 6615544 | 48.57 | 48 | 13 | 526 | 162163 | 170931 |
| P6 | NCA | 19190681 | 8828128 | 46.00 | 48 | 14 | 500 | 913361 | 913362 |
| P7 | NCA | 17459158 | 8034438 | 46.02 | 48 | 14 | 468 | 877164 | 1015325 |
| P8 | NCA | 19024646 | 8404508 | 44.18 | 45 | 14 | 491 | 198838 | 215431 |
| P9 | NCA | 21524023 | 9652271 | 44.84 | 44 | 14 | 481 | 188998 | 203271 |
| P10 | UA | 14347052 | 6895301 | 48.06 | 46 | 13 | 495 | 83452 | 86749 |
| P11 | UA | 12398284 | 6012950 | 48.50 | 49 | 13 | 511 | 493433 | 544012 |
| P12 | UA | 15827508 | 7623088 | 48.16 | 46 | 13 | 449 | 174357 | 184400 |
| P13 | UA | 12162319 | 5825420 | 47.90 | 45 | 13 | 499 | 232480 | 247642 |
| P14 | UA | 15562587 | 7514935 | 48.29 | 45 | 13 | 425 | 87691 | 91285 |
| P15 | UA | 17164104 | 8191082 | 47.72 | 43 | 13 | 391 | 83751 | 87405 |
| P16 | UA | 16797300 | 7954200 | 47.35 | 43 | 13 | 404 | 172516 | 182944 |
| P17 | UA | 12168250 | 5930675 | 48.74 | 49 | 13 | 545 | 112006 | 116466 |
| P18 | UA | 12495332 | 6044535 | 48.37 | 45 | 13 | 502 | 54364 | 55730 |
| P19 | AMI | 14999754 | 7236794 | 48.25 | 44 | 13 | 443 | 98316 | 102929 |
| P20 | AMI | 15276661 | 7377150 | 48.29 | 46 | 13 | 494 | 234729 | 250642 |
| P21 | AMI | 15821115 | 7633306 | 48.25 | 43 | 13 | 441 | 183293 | 194302 |
| P22 | AMI | 13684567 | 6583917 | 48.11 | 45 | 13 | 517 | 118714 | 124003 |
| P23 | AMI | 11277331 | 5403335 | 47.91 | 46 | 13 | 439 | 137878 | 144713 |
| P24 | AMI | 10664292 | 5124043 | 48.05 | 43 | 13 | 474 | 73616 | 76329 |
| P25 | AMI | 13311650 | 6392577 | 48.02 | 46 | 13 | 500 | 98355 | 103030 |
| P26 | AMI | 14715092 | 7117545 | 48.37 | 46 | 13 | 521 | 207406 | 220179 |
| P27 | AMI | 12699227 | 6106814 | 48.09 | 44 | 13 | 492 | 176903 | 186563 |
| P28 | AMI | 15916982 | 7714156 | 48.46 | 41 | 13 | 397 | 177087 | 187505 |
| P29 | AMI | 13562761 | 6558526 | 48.36 | 43 | 13 | 451 | 184236 | 195186 |
| P30 | AMI | 12795004 | 6157339 | 48.12 | 47 | 13 | 535 | 112073 | 116625 |
| P31 | AMI | 12537515 | 6044550 | 48.21 | 44 | 13 | 445 | 53136 | 54474 |
| P32 | AMI | 9982502 | 4821840 | 48.30 | 47 | 13 | 526 | 160407 | 168898 |

**Supporting Table S2 Frequent unique TCRβ CDR3 aa clonotypes in AMI patients**

| **TCRβ CDR 3aa** | **NCA(n=9)** | | **UA(n=9)** | | **AMI(n=14)** | | |
| --- | --- | --- | --- | --- | --- | --- | --- |
|  | Rate | Frequency | Rate | Frequency | Rate | Frequency | |
| CSVGRPSSSYNEQFF | 0.33 | 2.73E-05 | 0.44 | 4.87E-05 | 0.71 | 0.030 |  |
| CATSPRGAGRADYEQYF | 0.33 | 4.49E-06 | 0.44 | 5.53E-06 | 0.71 | 0.013 |  |
| CASTPSHNEQFF | 0.33 | 8.68E-06 | 0.44 | 1.44E-05 | 0.71 | 0.009 |  |
| CASSPLAVLADTQYF | 0.33 | 9.52E-06 | 0.44 | 1.13E-05 | 0.71 | 0.006 |  |
| CASAPGFTSRETQYF | 0.33 | 3.21E-06 | 0.44 | 7.89E-06 | 0.71 | 0.005 |  |
| CSVEGGQYYEQYF | 0.33 | 2.05E-06 | 0.44 | 5.16E-06 | 0.71 | 0.004 |  |
| CASNAGDEQYF | 0.33 | 5.16E-06 | 0.44 | 7.65E-06 | 0.71 | 0.004 |  |
| CASSPQGGYTYEQYF | 0.33 | 2.29E-06 | 0.44 | 2.47E-06 | 0.71 | 0.004 |  |
| CASSLAGGIAYEQYF | 0.33 | 5.59E-06 | 0.44 | 1.08E-05 | 0.71 | 0.003 |  |
| CSARGRETSGRAGTQYF | 0.33 | 2.11E-06 | 0.44 | 3.86E-06 | 0.71 | 0.003 |  |
| CASSVRPQGVSGNTIYF | 0.33 | 4.41E-06 | 0.44 | 8.35E-06 | 0.71 | 0.002 |  |
| CASSLWWGQNTEAFF | 0.33 | 2.36E-06 | 0.44 | 1.86E-06 | 0.71 | 0.002 |  |
| CASSRPEVRMGEQFF | 0.33 | 9.40E-07 | 0.44 | 5.52E-06 | 0.71 | 0.002 |  |
| CATSRDRDPVGTQYF | 0.33 | 2.86E-06 | 0.44 | 3.84E-06 | 0.71 | 0.002 |  |
| CASSFDEADTQYF | 0.33 | 2.71E-06 | 0.44 | 6.33E-06 | 0.71 | 0.002 |  |
| CASGSGQVIQETQYF | 0.33 | 1.85E-06 | 0.44 | 2.65E-06 | 0.71 | 0.002 |  |
| CASREHQLYEQYF | 0.33 | 1.52E-06 | 0.44 | 4.23E-06 | 0.71 | 0.002 |  |
| CASSGGLYNEQFF | 0.33 | 1.32E-06 | 0.56 | 1.99E-06 | 0.71 | 0.002 |  |
| CASSDSGQGYEQYF | 0.33 | 5.49E-07 | 0.44 | 4.11E-06 | 0.71 | 0.002 |  |
| CASSEASDYNEQFF | 0.33 | 1.12E-06 | 0.44 | 2.42E-06 | 0.71 | 0.001 |  |
| CASRGTSGRTNEQFF | 0.33 | 1.33E-06 | 0.44 | 2.01E-06 | 0.71 | 0.001 |  |
| CASSSTGLAVVTQYF | 0.33 | 1.95E-06 | 0.44 | 4.19E-06 | 0.71 | 0.001 |  |
| CASSQRAPLGDEQFF | 0.33 | 4.27E-07 | 0.44 | 4.04E-06 | 0.71 | 0.001 |  |
| CAIRGGLTNEQYF | 0.33 | 9.62E-07 | 0.44 | 1.82E-06 | 0.71 | 0.001 |  |
| CASSLSSLNEQFF | 0.33 | 1.87E-06 | 0.44 | 2.55E-06 | 0.79 | 0.001 |  |
| CASSSRPRDDSPLHF | 0.33 | 9.54E-07 | 0.44 | 3.21E-06 | 0.71 | 0.001 |  |
| CATSRHAGETQYF | 0.33 | 9.97E-07 | 0.44 | 1.59E-06 | 0.71 | 0.001 |  |
| CASKGQGSGTEAFF | 0.33 | 2.18E-07 | 0.44 | 2.84E-06 | 0.71 | 0.001 |  |

**Supporting Table S3 Frequent unique CDR3 aa clonotypes in UA patients**

| **CDR3 aa** | **NCA(n=9)** | | **UA(n=9)** | | | **AMI(n=14)** | | |
| --- | --- | --- | --- | --- | --- | --- | --- | --- |
|  | Rate | Frequency | Rate | | Frequency | | Rate | Frequency |
| CASSAGRETQYF | 0.00 | 0 | | 0.78 | 4.64E-07 | | 0.43 | 1.26E-05 |
| CASSLTSGLYNEQFF | 0.00 | 0 | | 0.78 | 5.59E-06 | | 0.21 | 9.51E-07 |
| CASSRTSGGYNEQFF | 0.00 | 0 | | 0.89 | 5.94E-07 | | 0.57 | 1.07E-06 |
| CASSPSGGQETQYF | 0.11 | 3.52E-08 | | 0.78 | 7.50E-06 | | 0.71 | 3.63E-06 |
| CASSSGQGYNEQFF | 0.22 | 6.22E-08 | | 0.78 | 4.84E-07 | | 0.64 | 6.59E-06 |
| CASSLRQGSTDTQYF | 0.22 | 7.77E-08 | | 0.78 | 1.58E-06 | | 0.36 | 2.29E-06 |
| CASSLFGGELFF | 0.22 | 6.59E-07 | | 0.78 | 4.51E-07 | | 0.50 | 6.43E-07 |
| CSARTSGYNEQFF | 0.22 | 1.17E-08 | | 0.78 | 3.01E-06 | | 0.21 | 2.27E-07 |
| CASSTSNQPQHF | 0.22 | 1.62E-08 | | 0.78 | 4.34E-06 | | 0.36 | 1.31E-07 |
| CASSLDGNNEQFF | 0.22 | 3.25E-08 | | 0.78 | 3.38E-06 | | 0.07 | 1.78E-07 |
| CASRGDNQPQHF | 0.22 | 2.27E-06 | | 0.89 | 4.02E-06 | | 0.36 | 6.05E-07 |
| CASSLSGGSYNEQFF | 0.33 | 2.78E-06 | | 0.78 | 2.60E-08 | | 0.71 | 2.19E-07 |
| CASRAYNEQFF | 0.33 | 2.76E-07 | | 0.78 | 3.92E-06 | | 0.29 | 3.61E-07 |
| CASSYSYNEQFF | 0.33 | 3.80E-08 | | 0.78 | 4.08E-06 | | 0.71 | 4.54E-06 |
| CASSLSGTGYEQYF | 0.33 | 4.55E-14 | | 0.78 | 1.04E-06 | | 0.43 | 3.88E-07 |
| CASSVTGGTDTQYF | 0.33 | 1.28E-08 | | 0.78 | 2.81E-06 | | 0.14 | 1.10E-08 |
| CASSYDSYNEQFF | 0.33 | 4.39E-07 | | 0.78 | 3.21E-06 | | 0.29 | 3.98E-07 |
| CASRGGSYNEQFF | 0.33 | 1.26E-07 | | 0.78 | 5.67E-07 | | 0.43 | 2.04E-07 |
| CAISEGYEQYF | 0.33 | 4.57E-07 | | 0.89 | 1.95E-04 | | 0.36 | 4.90E-06 |
| CASRDRGSTDTQYF | 0.33 | 5.73E-07 | | 0.78 | 5.49E-04 | | 0.71 | 4.80E-07 |
| CASSQTNQETQYF | 0.33 | 1.26E-07 | | 0.78 | 3.23E-06 | | 0.71 | 6.76E-07 |
| CASSLLAGGRNEQFF | 0.33 | 2.29E-07 | | 0.78 | 1.99E-06 | | 0.29 | 8.52E-07 |
| CASSSTGFGYTF | 0.33 | 2.13E-07 | | 0.78 | 8.27E-06 | | 0.57 | 8.93E-06 |
| CASSLGGGNNEQFF | 0.33 | 2.80E-08 | | 0.78 | 4.80E-06 | | 0.07 | 1.42E-08 |
| CASSSRRGETQYF | 0.33 | 1.88E-07 | | 0.78 | 6.07E-07 | | 0.14 | 1.13E-06 |
| CASRDTDTQYF | 0.33 | 5.48E-14 | | 0.78 | 2.87E-06 | | 0.43 | 4.88E-06 |
| CASSRGGNTGELFF | 0.33 | 1.46E-07 | | 0.78 | 8.62E-07 | | 0.64 | 1.14E-06 |
| CASSSGQGRETQYF | 0.33 | 1.27E-07 | | 0.78 | 1.67E-06 | | 0.50 | 1.32E-07 |
| CASSSGTSYNEQFF | 0.33 | 3.64E-13 | | 0.78 | 5.03E-07 | | 0.43 | 1.40E-07 |
| CASSPDRATDTQYF | 0.33 | 3.44E-14 | | 0.78 | 1.07E-07 | | 0.36 | 2.35E-07 |
